# Virtual ER, a Serious Game for Interprofessional Education to Enhance Teamwork in Medical and Nursing Undergraduates: Development and Evaluation Study

**DOI:** 10.2196/35269

**Published:** 2022-07-14

**Authors:** Janet Yuen-Ha Wong, Joanna Ko, Sujin Nam, Tyrone Kwok, Sheila Lam, Jessica Cheuk, Maggie Chan, Veronica Lam, Gordon T C Wong, Zoe L H Ng, Abraham Ka-Chung Wai

**Affiliations:** 1 School of Nursing Li Ka Shing Faculty of Medicine The University of Hong Kong Hong Kong Hong Kong; 2 Technology-Enriched Learning Initiative The University of Hong Kong Hong Kong Hong Kong; 3 Department of Anaesthesiology School of Clinical Medicine, Li Ka Shing Faculty of Medicine The University of Hong Kong Hong Kong Hong Kong; 4 Department of Emergency Medicine School of Clinical Medicine, Li Ka Shing Faculty of Medicine The University of Hong Kong Hong Kong Hong Kong; 5 Accident and Emergency Department The University of Hong Kong–Shenzhen Hospital Shenzhen China

**Keywords:** game, interprofessional education, teamwork, learning style, emergency medicine, emergency nursing

## Abstract

**Background:**

Engaging students in interprofessional education for higher order thinking and collaborative problem-solving skills is challenging. This study reports the development of Virtual ER, a serious game played on a virtual platform, and how it can be an innovative way for delivering interprofessional education to medical and nursing undergraduates.

**Objective:**

We report the development of a serious online game, Virtual ER, and evaluate its effect on teamwork enhancement and clinical competence. We also explore if Virtual ER can be an effective pedagogical tool to engage medical and nursing students with different learning styles.

**Methods:**

Virtual ER is a custom-made, learning outcome–driven, case-based web app. We developed a game performance scoring system with specific mechanisms to enhance serious gaming elements. Sixty-two students were recruited from our medical and nursing programs. They played the games in teams of 4 or 5, followed by an instructor-led debriefing for concept consolidation. Teamwork attitudes, as measured by the Human Factors Attitude Survey, were compared before and after the game. Learning style was measured with a modified Honey and Mumford learning style questionnaire.

**Results:**

Students were satisfied with Virtual ER (mean satisfaction score 5.44, SD 0.95, of a possible 7). Overall, Virtual ER enhanced teamwork attitude by 3.02 points (95% CI 1.15-4.88, *P*=.002). Students with higher scores as activists (estimate 9.09, 95% CI 5.17-13.02, *P*<.001) and pragmatists (estimate 5.69, 95% CI 1.18-10.20, *P*=.01) had a significantly higher degree of teamwork attitude enhancement, while students with higher scores as theorists and reflectors did not demonstrate significant changes. However, there was no difference in game performance scores between students with different learning styles.

**Conclusions:**

There was considerable teamwork enhancement after playing Virtual ER for interprofessional education, in particular for students who had activist or pragmatist learning styles. Serious online games have potential in interprofessional education for the development of 21st century life skills. Our findings also suggest that Virtual ER for interprofessional education delivery could be expanded locally and globally.

## Introduction

The emergency room (ER) is a unique clinical environment in which effective teamwork between doctors and nurses is necessary for prioritization of patient care, complex clinical decision-making, and safe yet efficient clinical practice [1]. However, these professionals are normally trained in their own professional silos with separate curricula and assessment schemes. The World Health Organization recommends interprofessional education (IPE) [2], which is increasingly recognized as a paramount tool to purposefully bring students from different disciplines together to learn teamwork, communications skills, and patient safety culture for collaboration in their future clinical practice.

IPE is challenging because of its demands for physical space, faculty time, timetable alignment, and student engagement [3]. However, its value is reflected in the successful enhancement of teamwork, as evidenced by many successful cases in previous reports [4-6]. With the teaching disruptions caused by the COVID-19 pandemic, the development of alternative methods of IPE delivery through online platforms is necessary to meet unmet demands.

Engaging students with collaborative problem-solving skills through higher-order thinking in online IPE is a challenge [7]. Online serious gaming is an innovative delivery mechanism for IPE. Unlike gamification, serious gaming should align with learning goals and drive students to engage in the game due to intrinsic factors, such as autonomy, relatedness, and competence, instead of extrinsic factors, such as points and badges [8]. There are diverse definitions of serious games in the literature. Bedwell and colleagues [9] empirically identified, in a systematic review, many attributes of serious games, including action language, assessment, conflicts and challenge, control, environment, game fiction, human interaction, immersion, and rules and goals. Indeed, the growing popularity of serious gaming in clinical education has been noted since 2010 [10,11]. Regarding teamwork and communications enhancement, there is also evidence showing that game-based surgical education training, with face-detection and head-tracking technology, can enhance nonverbal communications and facilitate teamwork [12]. There is also evidence that single-player screen-based teamwork training is feasible, and that it does not affect in-game performance [13]. However, there is a dearth of evaluation studies of serious online games for IPE to enhance teamwork among undergraduate students. Teamwork with members from different disciplines is particularly important in the ER to enhance diagnostic accuracy and efficiency and prevent medical errors in transitions of care. Therefore, IPE in undergraduate education is known to be essential for enabling an effective, collaborative practice eHealth-ready workforce for health care settings [14].

Clinical education today emphasizes student-centered approaches in teaching and learning. Although evidence supports the benefits of IPE, students’ attitudes toward IPE vary because of their highly diverse backgrounds, compounded by their different professional programs, different levels of clinical exposure, and different learning styles [15]. It is understood that different learning styles may affect student learning outcomes. However, there is a gap in evidence to understand how serious games in clinical IPE can be effective for students with different learning styles. Kolb’s learning cycle identified 4 learning styles: reflectors, theorists, activists, and pragmatists [16]. It has also been noted that face-to-face simulation-based activities are most effective for students who have a balanced learning style, including those who are theorists; in other words, they are effective for students who learn through abstract thinking, reflection, and carefully looking into problems from multiple perspectives [17]. On the other hand, game-based activities are most effective in activists and reflectors, who prefer concrete experience and reflective observation [18]. Therefore, awareness of learning styles will be helpful for clinical educators to modify and personalize gamification elements for effective strategies for student engagement in IPE.

In this study, we developed Virtual ER, an online serious game set in the context of an emergency room with specific learning goals. We evaluated the effect of Virtual ER on teamwork enhancement via a pilot study. Although we anticipated that this serious game would be an effective pedagogical tool to engage students in medicine and nursing, we were also interested in whether learning style, based on Kolb’s learning cycle [16], would be a key driving factor underlying the impact of serious games in IPE. We hypothesized that students who are activists and pragmatists would have greater score increases for teamwork attitude, as measured by the Human Factors Attitude Survey, compared to students who are reflectors or theorists (hypothesis 1). Moreover, we hypothesized that students who are activists and pragmatists would have higher scores for clinical competence compared with those who are reflectors and theorists (hypothesis 2).

We also examined whether students’ prior basic life support or advanced cardiac life training affected the primary outcomes and game performance scores. Our findings may lead to recommendations for pedagogical designs and best practices that are relevant to serious games for IPE with student-centered approaches.

## Methods

### Serious Game Development

#### Design Philosophy

Using constructivism as the underlying learning philosophy, our Virtual ER game provides a platform for cognitive, socio-cultural, and collaborative interactions for student learning [19]. Students as players are required to analyze scenarios and situations in the game and collaborate with others to select correct answers and demonstrate clinical competency.

#### Design Framework

**Figure 1:**
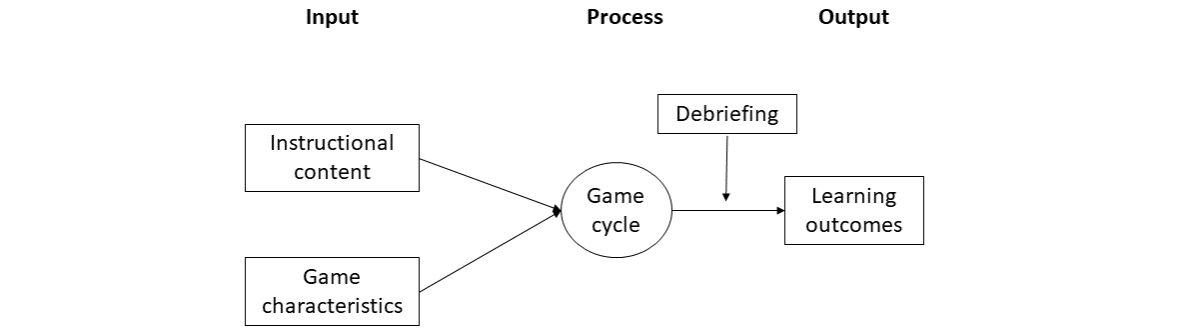
Illustration of the input-process-output model.

Virtual ER is a serious game designed based on the input-process-output (IPO) model, which is a tacit model of learning used in many studies [20-22]. Figure 1 illustrates the IPO model. First, the instructional program was designed to meet the learning goals and incorporated certain game features to facilitate students’ learning according to the goals. The learning goals included competency in history taking, physical assessment skills, diagnostic skills, alertness, treatment skills, clinical procedural skills, prioritization skills during emergency situations, professionalism, responsibility, and a caring attitude. Table 1 describes specific learning goals in a specific scenario used in the game. The scenario was introduced to the players as follows:

A 66-year-old man, Peter, was admitted to Accident & Emergency Department this morning due to intermittent chest pain for the last 24 hours. He was a chronic smoker and was previously diagnosed with hypertension and hyperlipidemia. Without any prior symptoms, he started to develop shortness of breath and decreased exercise tolerance in these few months. One hour ago, he suffered from severe chest pain with radiation to left shoulder. One tablet of glyceryl trinitrate was taken but effect was minimal. He then sought emergency medical care this morning due to pain persistence. Aspirin was given in the ambulance during transport after ruling out NKDA [no known drug allergies]. Peter’s wife, Jenny, accompanied upon admission. In this game, you are the nurse or physician responsible for Peter, who requires emergency care.

Second, the game enhances student communication and collaborative efforts by providing feedback on their engagement in the game and increasing their motivation to play. Engagement in game play, feedback from the game, and, most importantly, a debriefing session led to the achievement of learning goals and specific learning outcomes.

**Table 1:** Specific learning goals in a specific scenario used in the game.

Game stage	Learning goals	Game description	Student tasks (examples)	
			Medical students	Nursing students
Triage station	Competency in history taking skills: gathering relevant and accurate health information via history taking	Categorizing patients via the triage system	Be alert to the criteria of triage categorization Perform comprehensive assessment and history taking upon arrival Arrange appropriate investigation to rule out possible diagnoses	Be alert to patient severity and perform immediate medical management Assess pain level and determine if immediate treatment should be given at triage Obtain baseline vital signs upon patient arrival
Emergency cubicle	Competency in physical assessment skills, diagnostic skills, alertness, and treatment skills	Assessing patients’ condition in the emergency cubicle	Be alert to any signs of deterioration and perform prompt medical management Cooperate and communicate with team members to provide emergency care to the patient	Monitor patients closely and perform reassessments to obtain data for guiding further nursing management Be alert for any signs of deterioration and inform doctors if necessary
Resuscitation room	Competency in clinical procedural skills and prioritization skills during emergency situations	Providing and prioritizing appropriate treatment in an emergency situation	Perform emergency care efficiently according to advanced cardiac life support protocols Become familiarized with the use of different emergency medications during cardiopulmonary resuscitation Appropriately intubate patients in respiratory distress	Perform emergency care efficiently according to advanced cardiac life support protocols Cooperate and communicate with doctors to ensure emergency management can be promptly delivered. Assist doctors to perform intubation for patients with respiratory distress
Discharge to cardiac care unit	Competency in professionalism, responsibility, and a caring attitude	Preparing for a case transferal in emergency room	Consult corresponding department for taking over stabilized patients	Communicate with corresponding department about the condition of patients to guarantee continuous care can be arranged Prepare case transfer with valid documentation and proper functioning of medical support devices

#### Instructional Content

**Figure 2:**
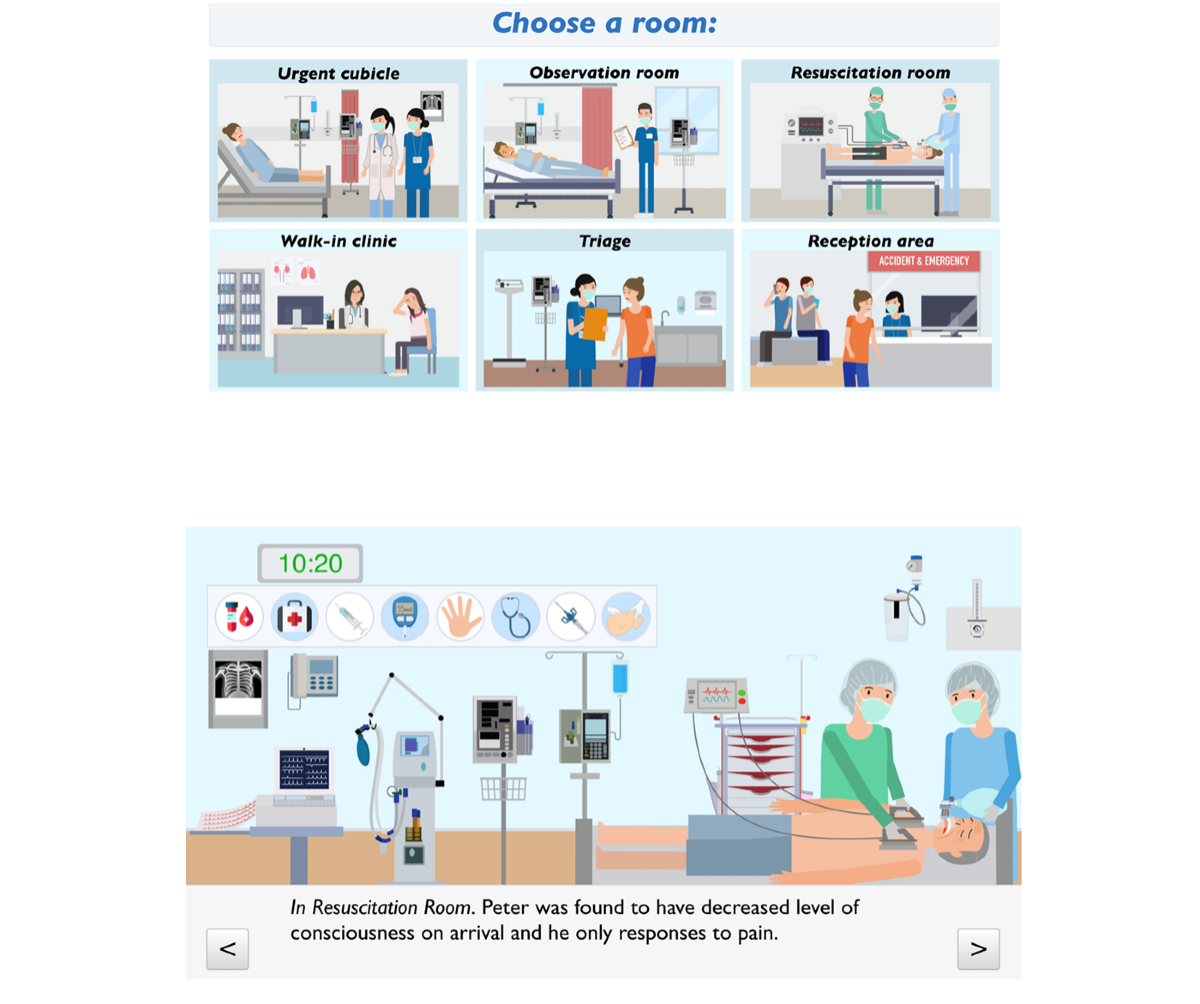
Screenshot of Virtual ER. The scenario starts at the triage center. Students can go into different rooms according to the scenario, including the resuscitation room.

Virtual ER begins with a virtual patient attending the emergency room on a Monday morning at 10 AM. There are 4 or 5 students playing doctor or nurse avatars with roles in triage, initial assessment, monitoring, resuscitation, and transfer of care. Virtual ER allows students to assess patients through their medical history, a physical examination (including vital signs), investigation (eg, electrocardiography, X-rays, CT scans, blood tests, and point-of-care tests), treatment (eg, prescriptions, drug administration, establishment of intravenous access, oxygen therapy, suturing, and wound care), and nursing processes, including caring (eg, phone calls, talking to patients to provide comfort, talking to relatives), charting, discharge, and transfer to different settings or rooms (Figure 2). The virtual patient’s condition deteriorates during the course of care and progresses to a need for resuscitation. Student players are required to provide urgent care to the patient according to different priorities. When we set up the challenges for students, some questions were designed particularly for medical students and some questions for nursing students. Performance scores were allocated to the teams to complete decision-making or behavioral tasks accurately, safely, and properly. Table 1 shows the students’ responsibilities in managing the virtual patient.

#### Game Characteristics and Performance Scoring System

**Figure 3:**
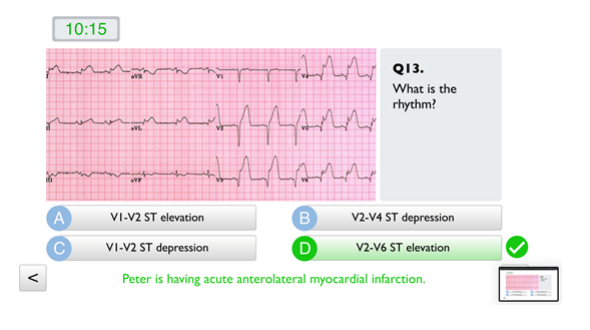
Screenshot of Virtual ER. The question is about interpreting electrocardiogram results. ER: Emergency room. Q3: Question 3. ST: represents wave on an electrocardiogram. V1-V6: represents electrocardiogram leads.

Virtual ER is a custom-made web app built with Tumult Hype (Tumult Inc), a tool for creating interactive web content. The performance scoring system used three mechanisms to enhance student learning: (1) the students’ first response to each question (ie, their selected actions and choices in the game) was stored in the system and allocated to the team. This facilitated teachers’ performance analysis and subsequent debriefing by the end of the class. It also left the students unaware of their performance during game play, leaving them to discover their number of mistakes at the end of the game [23]. Figure 3 shows a question about electrocardiogram results. (2) The students were allowed to go back to prior questions and select other actions or choices, but this did not lead to any change in the score assigned based on the already attempted question. This allowed students to think carefully before answering questions and enhanced face-to-face interpersonal discussion among team members [24]. This rule was emphasized at the beginning of the game. (3) Incorrect actions and choices led to score deductions, to drive students to rationalize each decision and discourage answering randomly. This penalty system was designed to discourage undesirable behaviors in a learning environment [25].

The scoring system was implemented using JavaScript inside the Tumult Hype application. The students’ performance data was stored on the Firebase cloud server (Google LLC). These data are downloadable for analysis.

#### Procedures

**Figure 4:**
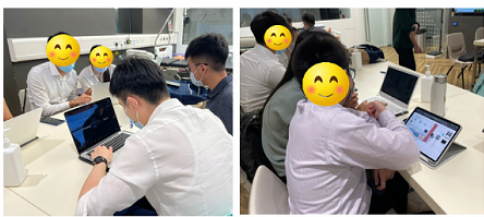
Images of student discussion while playing Virtual ER. ER: Emergency room.

The learning goal of Virtual ER was to develop a sense of teamwork among medical and nursing students. Students were assigned into interdisciplinary groups with 4 to 5 students each. They accessed Virtual ER online simultaneously on laptops. Before the game started, all students were briefed on the educational aims and objectives and provided game orientation. Written informed consent for evaluation was then sought. The students were invited to complete a 10-minute pretest questionnaire. Instructions for downloading the online game were also provided. The students were given 30 minutes to play the game with their teammates. During the game, teachers encouraged the students to share their domain knowledge and to discuss the best clinical decisions for the virtual patient. All their decisions or behaviors during the game were evaluated systematically for the clinical competence they showed regarding patient management. At the end of the game, all the groups were invited to join the debriefing as a whole class. Figure 4 shows student peers discussing the Virtual ER game. Emergency medicine and nursing teachers cotaught during the debriefing, with the dynamics and experiences of different groups linked to teamwork and clinical competency in handling the virtual patient in the scenario. Upon completion of the debriefing, the students were invited to fill out a posttest questionnaire, which took around 10 minutes to complete. The whole session lasted for about 2 hours.

### Participants and Setting

All students in the emergency clerkship course and all nursing undergraduates in the emergency nursing-care course of a university were invited to participate via email and e-flyer. The pilot study took place between August 2021 and November 2021. Sixty-two final-year students participated. They were well equipped with necessary clinical knowledge and skills and had received relevant emergency care training prior to this study.

### Evaluation Measurements

#### Attitudes Toward Teamwork (Primary Outcome)

We assessed attitudinal shifts to team behavior with the Human Factors Attitude Survey (HFAS), which was developed by the University of Texas and the National Aeronautics and Space Administration [26]. The HFAS has 23 items with good internal reliability (Cronbach α=.89) and has been tested in Hong Kong [27]. All questions were reviewed by expert doctors and nurses for content and face validity to ensure that the survey was subjectively viewed as covering the concepts it measured. Participants were asked to indicate their agreement with each question on a 5-point Likert scale (from 1, indicating “strongly disagree,” to 5, “strongly agree”). Some examples of questions include the following: “my performance is not adversely affected by working with an inexperienced or less capable team member,” “prior to the procedure, it is important for all team members to be familiar with the tasks and responsibilities of the other members of the team,” and “my ability to detect adverse situations has a direct relationship to the quality of decisions I make.” This scale was used in pre- and posttest questionnaires. The total score ranged between 23 and 115.

Clinical competencies were assessed for handling patients in the case scenarios. Students were provided with a performance score upon completion of a case scenario. Marks were given for correct answers in the game and marks were deducted for incorrect answers. The total possible score for each case was 41.

#### Learning Style

A modified version of the Honey and Mumford learning style questionnaire was used [28]. It contained 13 items with responses on a 5-point Likert scale (1 indicating “strongly disagree,” 2 indicating “disagree,” 3 indicating “neutral,” 4 indicating “agree,” and 5 indicating “strongly agree”). It was tested and found to have satisfactory Cronbach α values, ranging from α=.593 to α=.786 for the 4 different types of learners: reflectors, theorists, activists, and pragmatists. The higher the scores for a specific learning style, the more likely it was that the students adopted that learning style. Learning styles were assessed in the pretest questionnaire.

#### Demographics

Demographics included sex, study program, and clinical part-time job experience. Information on additional basic life support and advanced cardiovascular life support training was also gathered with the pretest questionnaire.

#### Virtual ER Game Satisfaction

One question was included in the posttest questionnaire to assess game satisfaction on a 7-point Likert scale, ranging from “not at all satisfied” to “extremely satisfied.” The question was “How satisfied are you with the game and tutorial?”

### Data Management and Analysis

Data were collected with an online questionnaire that used the Qualtrics platform (Qualtrics). Descriptive statistics were used to describe the students’ characteristics. Quantitative data were entered into SPSS (IBM Co). Changes in teamwork attitude over time were assessed with a paired *t* test (2-tailed). We used linear mixed effect modeling (LMM) to evaluate whether students’ learning styles and prior training in basic life support or advanced cardiovascular life support had any effect on changes in teamwork attitude. LMM has been used for the analysis of between-participant data, including both fixed effects and random effects [29]. This was helpful for the robustness of the data analysis, in particular, for evaluating the effects of differences in the study population in terms of learning styles and a priori training and clinical experience.

### Ethical Approval

Ethical approval was obtained from the University of Hong Kong/Hospital Authority Hong Kong West Cluster Joint Institutional Review Board (UW 21-302). A signed online consent form was obtained before data collection. During the informed consent process, an information sheet was provided that included information on the credentials and affiliations of the researchers and the purpose of the evaluation.

## Results

Of 62 students, 34 (54%) were male and 47 (76%) were studying medicine. There were 38 students (61%) who had attended basic life support training and 35 (56%) students who had attended advanced cardiovascular life support training before joining the Virtual ER game workshop. There were 20 (32%) activists, 19 (31%) pragmatists, 4 (7%) reflectors, and 3 (5%) theorists. There were 16 students (26%) with a combination of 2, 3, or 4 styles. Mean scores on the Honey and Mumford questionnaire for the activists, theorists, reflectors, and pragmatists were 3.76 (SD 0.49), 3.47 (SD 0.52), 3.24 (SD 0.62), and 3.71 (SD 0.47), respectively.

Overall, Virtual ER enhanced teamwork attitudes. The pretest HFAS score was 91.26 (SD 8.75) and the posttest score was 94.27 (SD 10.46), representing a 3.02-point increase (95% CI 1.15-4.88, *P*=.002). We used a mixed effect model to further investigate the effect of learning style on teamwork attitude enhancement (Table 2 shows the results). As we hypothesized (hypothesis 1), students who had higher scores as activists (estimate 9.09, 95% CI 5.17-13.02, *P*<.001) and pragmatists (estimate 5.69, 95% CI 1.18-10.20, *P*=.01) had significantly higher teamwork attitude enhancement, while students with higher scores as theorists and reflectors showed no significant change. Regarding the Virtual ER game performance scores, there was no difference among students with different learning styles. Therefore, hypothesis 2 was not supported.

Students with prior experience in basic life support showed no significant difference in teamwork enhancement or Virtual ER game performance. However, students with prior advanced cardiovascular life support training showed a decrease in teamwork attitude between baseline and after playing the game (estimate –4.86, 95% CI –9.13 to –0.59, *P*=.03).

In general, students were satisfied with Virtual ER, with a game satisfaction score of mean 5.44 (SD 0.95) points of a possible 7. Of the 62 students, 2 (3%) rated their experience with the app “not satisfied,” 6 (10%) rated it “neutral,” 25 (40%) rated it “satisfied,” 21 (34%) rated it “very satisfied,” and 8 (13%) rated it “extremely satisfied.”

**Table 2:** Effect of learning style on teamwork enhancement and performance scores.

		Teamwork enhancement				Virtual ER^a^ game performance			
Outcomes		Estimate	95% CI	*P* value	Estimate			95% CI	*P* value
**Learning style**									
	Activist	9.09	5.17 to 13.02	<.001	–5.75		14.77 to 3.26		.21
	Theorist	1.81	–2.46 to 6.08	.41	–8.17		–16.52 to 0.17		.06
	Reflector	2.49	–1.04 to 6.02	.16	–6.42		–13.42 to 0.58		.07
	Pragmatist	5.69	1.18 to 10.20	.01	–5.03		–14.44 to 4.38		.29
**Prior experience**									
	Basic life support	–2.84	–9.48 to 1.13	.12	–1.70		–10.77 to 7.36		.71
	Advanced cardiovascular life support	–4.86	–9.13 to –0.59	.03	3.87		–4.99 to 12.73		.39

^a^ER: Emergency room.

## Discussion

### Principal Findings

Using a serious game approach for clinical education is an emerging field, and our study is among the first to report on the development of a custom-made, ad-hoc, collaborative game, Virtual ER, and evaluate it for its ability to enhance teamwork among medical and nursing undergraduate students. More importantly, we demonstrate that Virtual ER enhanced teamwork in students with a small but statistically significant effect, and that it was particularly and significantly effective in students who had more activist or pragmatist personalities. This finding supports our hypothesis 1. Compared with our previous study, which examined face-to-face interprofessional clinical simulation [5], Virtual ER was as effective at enhancing teamwork among medical and nursing students as clinical simulation. In addition, Virtual ER was widely accepted by the medical and nursing students and was rated highly for satisfaction. Apart from establishing the validity of serious games in interprofessional education, it was worthwhile to provide an enjoyable and motivating environment for students.

Using serious games for interprofessional learning outcomes is not new. Previous studies have used card or board games [30-33] to demonstrate positive outcomes for teamwork, communication, and interprofessional roles. Their results are consistent with our current findings on teamwork enhancement as a primary outcome. However, our study is innovative in terms of the pedagogical material that it included as part of the routine curriculum, giving students the opportunity to learn not only during the game, but also during the debriefing. The use of a virtual platform also eased the demand for physical space for huge class sizes. Medical students and nursing students are taught about emergency and urgent care at different years of their education. When they played Virtual ER, they were immersed in an ER environment that allowed them to perform triage, assess patients, formulate differential diagnoses, request investigations, and offer treatments. They were also able to recognize clinical deterioration and initiate resuscitation. During the debriefing, the teachers facilitated discussion with the students about managing patients and their shared experiences in handling patients in the ER. The debriefing provided opportunities for students to reflect, critique, and correct what they learned to further enhance their clinical competencies [34].

Another innovative aspect of this study was the assessment of the students’ learning styles, which helped to determine whether there were any mismatches between teaching modes and learning styles. We found that the effect of Virtual ER on teamwork was much stronger in students who had more activist and pragmatist personality characteristics. In other words, they were more engaged in the game and gained more benefits in regards to teamwork enhancement from the game. Activists prefer learning by experience and taking direct action, while pragmatists like to experiment, see the relevance of their work, and adopt problem-solving approaches during learning [16]. Our game presented an immersive scenario in which a virtual patient was admitted to an ER with chest pain. The students discussed the case and chose the best investigation methods and treatments to save the patient’s life. The problem-based learning structure of the game thus closely matched the preferences of activist and pragmatist learners. This also explains the positive primary outcome of this study: the game significantly enhanced teamwork. However, our results failed to support hypothesis 2, which was that students who were activists or pragmatists would have high game performance scores. This finding is in line with other studies, which have shown a lack of a direct relationship between learning experience and academic performance in medical students [35-37]. It is possible that learning style can engage students in learning in serious games; however, it might not be a predictor of academic success. Criticism of the idea that adapting to individual learning styles can provide better academic performance can be found as early as 2004 [38]. In Virtual ER, students who tend to construct their own knowledge might have relied heavily on the debriefing, especially the opportunity for reflection, critique, and correction. Therefore, the game elements of Virtual ER and its design may not be adaptive enough to facilitate student learning. Alternative approaches to adapting serious games to cater to different students have recently been introduced, such as Felder-Silverman’s model [39], which combined adaptive game-element designs for collaboration, gamification, and content interactions [40]. Although that study was designed for individual students interacting with the system, it would be a worthwhile approach to the further expansion of IPE among medical and nursing students in the future.

We found that learning styles were associated with teamwork enhancement but not clinical competency. This implies that individual students with different learning styles could find their own way to a more adaptive approach to learning. The students were encouraged to discuss the best clinical decisions for the virtual patient during class, and this discussion may have helped the students pick the correct answers and increase their performance. This may be beneficial, because as medical and nursing educators, we should provide a positive learning environment to facilitate individual students to develop their own learning approaches to optimize their academic performance. Nevertheless, in this study, students with prior advanced cardiovascular life support training showed a decrease in scores for teamwork attitude between baseline and after using the game. It is unclear whether students equipped with more advanced clinical knowledge tended to have more confidence and to work more on their own and therefore did not value the teamwork enhancement that Virtual ER made possible. This finding is consistent with a past study that showed that students had increased confidence in their own knowledge after basic life support training [41]. However, in the 21st century, in addition to academic and literacy skills, interprofessional collaborative practice is essential in the health care system. IPE is an important, innovative strategy for mitigating the global health workforce crisis, which has been reported by the World Health Organization [2]. Teamwork and collaboration skills cannot be built without engaging in practice and attitudinal changes. Therefore, educators should be creative in designing innovative pedagogical methods to match students’ learning styles and engage them in learning essential “soft” skills, including teamwork, collaboration, communication, and leadership, for their personal and professional mastery. Serious games have potential for developing such attributes. Our findings demonstrate that our serious game positively increased teamwork attitudes and that this self-developed game should be a useful basis for further prospective expansion and enhancement. In the future, we would like to add learning goals on teamwork training. Furthermore, it will be possible to develop Virtual ER into an online platform, enabling IPE delivery regionally or globally.

### Study Limitations

A few limitations weaken the generalizability of our results. First, this was a cohort study of 62 students without a comparison group, and the significant positive outcome may be attributable to the content of the game scenario, rather than to serious gaming in general. Therefore, a randomized controlled trial should be conducted in the future. We also did not examine the long-term effect of the serious game on teamwork enhancement, as our study was only focused on a single intervention. Future studies are needed to explore changes in teamwork attitude over a longer period, especially after graduation. Additionally, emergency medicine education is spread out in our medical curriculum, and the nursing students also had experience from part-time jobs as assistants in the health care system. Therefore, it is possible that some students may have already been exposed to some of the clinical conditions in the game, while others had not been. In this study, the majority of participants were medical students, and the effects of Virtual ER may have been unique in these students. Although we examined whether previous training in basic life support or advanced cardiovascular life support was associated with the study outcomes, it was still unclear whether a priori clinical experience affected some of the potential learning effects, nor was it clear how strong this effect might have been. In a future study with a larger sample size, we hope to perform a subgroup analysis to understand this relationship. Lastly, a future study could use a qualitative design to examine student satisfaction with the game to further improve it.

### Conclusion

This study reports on the development of Virtual ER and evaluates its effect on teamwork enhancement in IPE and on clinical competency in handling patients with cardiovascular problems. Our findings demonstrate that this serious game had a positive effect on teamwork enhancement, in particular for students who had activist and pragmatist learning styles. We suggest that serious online games are a potential tool for the development of IPE and the fostering of 21st century life skills. Owing to its use of a virtual platform, this game might also be an innovative way to deliver IPE locally and globally.

## Abbreviations

ER: emergency room
HFAS: Human Factors Attitude Survey
IPE: interprofessional education
IPO: input-process-output
LMM: linear mixed effect modeling
NKDA: no known drug allergies
